# Association Between Dietary Polyphenol Intake and Semen Quality: Insights from the FERTINUTS Study

**DOI:** 10.3390/nu17172785

**Published:** 2025-08-27

**Authors:** Hamza Mostafa, Javier Mateu-Fabregat, Asmae Benchohra, Nil Novau-Ferré, Laura Panisello, Mònica Bulló

**Affiliations:** 1 Nutrition and Metabolic Health Research Group, Department of Biochemistry and Biotechnology, Rovira i Virgili University (URV), 43201 Reus, Spain; javier.mateu@urv.cat (J.M.-F.); asmae.benchohra@estudiants.urv.cat (A.B.); nil.novau@urv.cat (N.N.-F.); laura.panisello@urv.cat (L.P.); 2 Institute of Health Pere Virgili (IISPV), 43201 Reus, Spain; 3 Center of Environmental, Food and Toxicological Technology (TecnATox), Rovira i Virgili University, 43201 Reus, Spain; 4 CIBER Physiology of Obesity and Nutrition (CIBEROBN), Carlos III Health Institute, 28029 Madrid, Spain

**Keywords:** male infertility, polyphenol, nutrition, semen quality

## Abstract

Background/Objectives: Low semen quality and male infertility are critical global health issues. Emerging research highlights that nutritional factors could play a significant role in determining reproductive outcomes. Understanding and optimizing these dietary influences, including the role of polyphenols, is crucial for developing targeted strategies to improve male fertility. We aimed to explore the relationship between the intake of different classes of polyphenols and semen quality indicators in a cohort of healthy young males. Methods: This is a secondary analysis involving 106 male individuals, aged 18–35 years, from the FERTINUTS trial. Dietary intake was assessed using 3-day dietary records, and semen quality parameters were analyzed. Multivariable linear regression analysis was employed to evaluate the associations between dietary polyphenol consumption and semen quality indicators. Results: Our findings revealed both positive and negative associations between polyphenol consumption and sperm morphology parameters. A higher intake of total polyphenols was associated with a lower percentage of abnormalities in sperm heads but a higher rate of abnormalities in the principal piece. Similar results were observed for lignan and flavonoid intake. Additionally, a higher intake of flavonoids was also associated with a greater percentage of normal sperm forms. In contrast, a higher dietary intake of stilbenes was associated with a higher percentage of combined abnormalities. Conclusions: Higher intake of polyphenols, particularly flavonoids and lignans, was associated with improved sperm head morphology but also with increased tail abnormalities, although no associations with motility or vitality were observed. These results suggest that specific polyphenol classes may have both beneficial and adverse effects on sperm structure, warranting consideration of compound type and dosage in dietary recommendations. Further studies are needed to determine whether these morphological changes impact fertilization outcomes and reproductive potential.

## 1. Introduction

According to the World Health Organization (WHO), infertility is defined as the inability to achieve pregnancy after 12 months or more of regular, unprotected sexual intercourse [1]. Male infertility contributes to approximately 30% of infertility cases, with global prevalence estimates ranging from 9% to 15% [2]. Regional data indicate that the Eastern Mediterranean has one of the lowest lifetime prevalence rates, around 10% [1].

While genetic and endocrine disorders are well-established contributors to male infertility, semen quality is also strongly influenced by modifiable lifestyle and environmental factors. These include diet, physical activity, smoking, alcohol consumption, obesity, psychological stress, and exposure to air pollution and environmental toxins such as heavy metals [3,4,5]. Adherence to healthy dietary patterns such as the Mediterranean and the Dietary Approaches to Stop Hypertension (DASH) diets which emphasizes fruits and vegetables consumption, moderate intake of low-fat dairy products, low consumption of animal protein, and high intake of plant-based protein from legumes and nuts has been linked to improved semen quality in a recent systematic review and meta-analysis of 1244 subjects across six studies [6]. Additionally, cross-sectional studies among young men in Greek and Italian populations have associated the Mediterranean diet with better sperm quality [7,8].

Polyphenols, naturally occurring compounds in plant-based foods like fruits and vegetables, and particularly abundant in the Mediterranean diet, are classified into several classes based on the number of phenolic rings and functional groups, such as phenolic acids, flavonoids, stilbenes, and lignans, which helps in understanding their bioavailability and health benefits [9,10]. Polyphenols have attracted attention due to their antioxidant and anti-inflammatory properties. While they are best known for their benefits in cardiovascular health, neurodegenerative diseases, and reducing all-cause mortality [11,12,13], they have also emerged as potential modulators of reproductive function [14]. Evidence from preclinical studies suggests that these compounds may influence sperm quality by reducing oxidative stress, enhancing mitochondrial function, preserving DNA integrity, and increasing motility [15]. However, findings from human studies remain limited and inconsistent, with differences likely depending on polyphenol type, dose, and individual biological responses [16,17,18]. Despite growing interest, most research has focused on broad dietary patterns or single polyphenol compounds, often overlooking class-specific effects on sperm morphology and function. Since different polyphenol classes may vary in their bioavailability, antioxidant properties and biological activities [19,20], they may differentially influence sperm morphology and function. To date, no study has systematically investigated whether subclass-specific differences in polyphenol intake are associated with distinct semen quality outcomes in humans, leaving an important knowledge gap. In this context, we conducted a cross-sectional analysis within the FERTINUTS trial to explore associations between the intake of different polyphenol classes and detailed semen quality parameters in healthy men. We hypothesized that greater intake of specific polyphenol classes would be differentially associated with favorable semen quality outcomes, reflecting the distinct antioxidant capacities and biological activities of each class.

## 2. Materials and Methods

### 2.1. Study Design

This study is a cross-sectional secondary analysis using baseline data from the FERTINUTS trial, a 14-week randomized controlled study conducted on healthy men aged 18–35 years. Participants were non-smokers, free of reproductive disorders and chronic illness, and did not use multivitamins or other antioxidant supplements. Detailed information about the trial can be found elsewhere [21].

### 2.2. Nutritional Data

To estimate the participants’ dietary intake, food consumption was recorded using 3-day dietary records, including two weekdays and one weekend day. Total energy, macronutrient, and micronutrient intake were estimated using the Spanish composition tables [22]. Polyphenol intake in mg/day was estimated using “Phenol Explorer version 3.6”, with polyphenol values expressed in mg/100 mL for liquids and mg/100 g for solid foods. For each polyphenol subclass (flavonoids, phenolic acids, lignans, stilbens, and others), the polyphenol content per 100 mL or 100 g of each food was multiplied by the average amount consumed over the 3-day period, then divided by 100 to express the result in mg/day. Total polyphenol intake was calculated as the sum across all subclasses, including flavonoids, phenolic acids, lignans, stilbenes, and other minor polyphenols.

### 2.3. Semen Quality Assessment

Semen samples were obtained after three days of sexual abstinence. Analyses of the freshly collected samples were performed within a maximum timeframe of 60 min post-collection as previously described [21]. The standard semen parameters, volume, pH, sperm motility, vitality, and morphology, were evaluated on fresh samples. The total sperm count and concentration were quantified using a 100 μm-deep hemocytometer chamber (Neubauer chamber), employing bright-field optics at ×400 magnification. Sperm motility was analyzed under a light microscope at ×400 magnification, categorizing sperm into three classifications: (1) progressive motility, (2) non-progressive motility, or (3) immobility. The motility was then represented as a percentage of total motility (comprising both progressive and non-progressive motility). Sperm vitality was assessed by examining the integrity of the sperm plasma membrane utilizing eosin-nigrosine at ×1000 magnification. Sperm morphology was evaluated on semen smears stained with Hemacolor (Millipore, Burlington, MA, USA) at ×1000 magnification, distinguishing normal sperm from defects in the head, midpiece, or principal piece (individually or combined). Morphology was reported as a percentage of normal forms. These parameters were evaluated following the guidelines set by the 2010 WHO [23].

### 2.4. Blood Analysis and Anthropometric Measurements

Weight (TANITA TBF-300, Tanita Corporation, Tokyo, Japan), height, body mass index (BMI), waist circumference, blood pressure (Omron HEM-705CP, Omron Healthcare Co., Ltd., Kyoto, Japan), general medical, and reproductive data were recorded [21]. Blood samples were collected during the visit after a twelve-hour fast. Fasting plasma glucose, total cholesterol, HDL and LDL cholesterol, triglycerides, insulin, C-reactive protein and folate were measured using standardized enzymatic automated systems.

### 2.5. Statistical Analysis

The characteristics of the study participants are presented as the mean (SD) or as the median [P25–P75] depending on the distribution of each variable. Polyphenol levels and sperm quality parameters were log-transformed to approximate normal distributions. Missing values for covariates were imputed using median values. Multivariable linear regression analyses were conducted to assess the cross-sectional associations between total dietary polyphenol intake and its specific subtypes and semen quality parameters. Participants were also categorized into tertiles based on their total polyphenols and subtype-specific polyphenol consumption. To assess trends across tertiles, the median values of each tertile were modelled as a continuous variable in the multivariable models. Results are reported as β coefficients with 95% confidence intervals (CIs), using the lowest tertile as the reference category. The models were adjusted for potential confounders, including age, BMI, plasma cholesterol level, total energy intake, and the intake of protein, fiber, carbohydrates, alcohol and fatty acids (monounsaturated, polyunsaturated, and saturated). All statistical analyses were performed using R version 4.2.2 (R Foundation for Statistical Computing, Boston, MA, USA).

## 3. Results

Of the 244 participants assessed for eligibility, 106 met the inclusion criteria for participating in the study and had complete data available for analysis. The participants had a mean age of 24.6 ± 4.7 years, a mean BMI of 23.8 ± 3.13 kg/m^2^, and an average total energy intake of 2495.08 ± 613.61 kcal/day. The median of total polyphenol intake was 404.7 mg/day [296.0–529.2] (Table 1).

**Figure 1:**
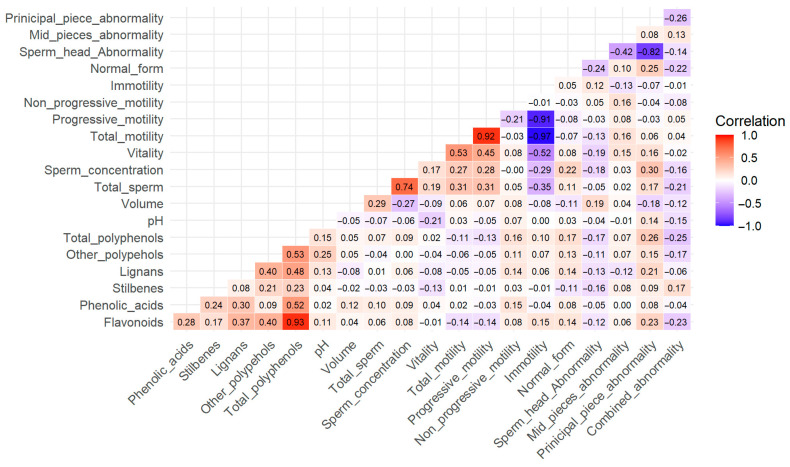
Association between different polyphenol subclasses and semen parameters. Spearman’s rank correlation coefficient is shown for each correlation.

Table 2 and Supplementary Table S1 show the associations between the intake of different polyphenol classes and semen quality parameters. No associations were observed between total polyphenol intake and pH, volume, sperm concentration, vitality or motility. However, sperm morphology showed statistically significant associations with polyphenol intake. Specifically, higher total polyphenol intake was associated with a lower percentage of sperm head abnormalities (β: −0.084; 95% CI: −0.153, −0.014; *p*-value = 0.02) but a higher rate of abnormalities in the principal piece (β: 0.375; 95% CI: 0.123, 0.627; *p*-value = 0.004). Regarding polyphenol subclasses, similar trends across tertiles were observed for lignan and flavonoid intake, both of which were inversely associated with sperm head abnormalities (lignan: β: −0.075; 95% CI: –0.147, –0.003; *P*-trend = 0.04; flavonoid: β: −0.078; 95% CI: –0.142, –0.014; *P*-trend = 0.01) and positively associated with abnormalities in the principal piece (lignan: β: 0.277; 95% CI: 0.011, 0.543; *P*-trend = 0.04; flavonoid: β: 0.305; 95% CI: 0.069, 0.540; *P*-trend = 0.01). Additionally, higher flavonoid intake was associated with a greater percentage of normal sperm forms (β: 0.088; 95% CI: 0.0, 0.175; *P*-trend = 0.049). In contrast, individuals within the highest intake of stilbenes had an increased percentage of combined sperm abnormalities compared to those in the lowest tertile (β: 0.175; 95% CI: 0.018, 0.322; *P*-trend = 0.01). Overall, participants with a higher dietary intake of total polyphenols, flavonoids, and lignans were associated with fewer head abnormalities but more abnormalities at the principal piece. Additionally, participants in the highest tertile of flavonoid intake showed a higher proportion of normal forms, while higher stilbene intake was associated with a lower frequency of combined sperm abnormalities. A heatmap of Spearman correlations between polyphenol subclasses and semen parameters is provided (Figure 1).

## 4. Discussion

The findings of this cross-sectional analysis contribute to the emerging literature on dietary polyphenol intake and male reproductive health by investigating their association with semen quality parameters in healthy men. Our findings suggest that while total and subclass-specific polyphenol intake, such as lignans and flavonoids, were not significantly associated with general semen parameters, including pH, volume, concentration, vitality, or motility, they were associated with sperm morphology. Specifically, higher intake of total polyphenol, lignans and flavonoids was associated with fewer head abnormalities, a finding that may be consistent with improved DNA integrity and acrosome function, yet higher tail abnormalities, which did not appear to impair sperm mobility.

These results offer partial support to existing biological hypotheses and experimental evidence. Preclinical studies, including in vitro and animal models, have consistently demonstrated the protective effects of polyphenols on sperm quality through antioxidant and anti-inflammatory mechanisms. For instance, in vitro experiments on human sperm have shown protective effects of polyphenol-rich extracts against oxidative stress, suggesting potential applications for fertility treatments and sperm storage [15]. However, the results are not universally consistent; as another in vitro study on stallion semen found no significant benefits, indicating that effects may vary depending on species, compound type, or concentration used [24]. Animal models generally report beneficial outcomes from dietary polyphenols, including improved sperm count, motility, and morphology [25,26].

Translating these preclinical findings into human populations, however, remains complex. A recent systematic review and meta-analysis, including four studies with 875 participants, found that nut consumption, rich in polyphenols, improved certain sperm parameters such as motility, vitality, and morphology, but not concentration, possibly by enhancing antioxidant capacity and modulating glucose metabolism through enzyme inhibition [16]. Similarly, a pilot trial using a resveratrol-based supplement, a natural stilbene, in 20 infertile men observed improvements in concentration and motility after several months, potentially due to enhanced mitochondrial function [27]. Furthermore, in an RCT involving infertile patients, daily consumption of walnuts, known for their high polyphenol content and other nutrients, was associated with significant improvements in sperm motility and concentration after three months of supplementation [28]. Taken together, these limited clinical findings suggest a potential beneficial role of polyphenols in improving semen quality among infertile men. However, given the small number and scale of available trials, further well-designed studies are needed before firm conclusions can be drawn.

Nonetheless, Ferramosca et al. noted that the effects of polyphenols may be dose-dependent [17]. This dual behavior is exemplified by quercetin, a flavonoid that acts as an antioxidant at low doses but may exert pro-oxidant effects at higher concentrations or in oxidative environments [18]. Additionally, a large cross-sectional study in China investigated the relationship between nine phytoestrogens, plant-derived polyphenols with estrogen-like activity, and found that higher semen levels of one lignan, secoisolariciresinol, and one isoflavone, genistein, were associated with lower sperm concentration, total sperm count, and motility [29]. In contrast, the level of naringenin, a flavanone, was positively associated with progressive and total motility [29].

Although our study population had relatively low stilbene intake, a statistically significant association was observed with a higher percentage of sperm combined abnormalities. Current literature suggests that resveratrol, a natural stilbene, may support male fertility by improving semen parameters and protecting sperm from oxidative damage, particularly at low concentrations (~6–15 μM), while higher doses (~100 μM or more) may exert cytotoxic effects on sperm function [30]. However, in vivo evidence on resveratrol’s effectiveness in improving semen parameters remains limited and inconsistent, underscoring the need for further well-designed clinical studies [31].

Notably, the observed pattern (fewer head abnormalities but more tail abnormalities with higher intake of selected polyphenol classes) has to date, no established mechanistic explanation in humans. Polyphenols may differentially influence sperm compartments through redox modulation and signaling pathways, but current evidence is indirect. In cadmium-exposed male mice, resveratrol attenuated sperm morphology defects and reduced overexpression of epidermal growth factor receptor (EGFR)-related signaling proteins, suggesting that polyphenols can modulate protein expression and mitigate oxidative/inflammatory stress in the reproductive tract [32]. However, these data do not confirm a head-specific benefit and tail-specific risk in humans. Future studies should incorporate CASA-derived kinematics analysis, mitochondrial function, and proteomics analysis to elucidate whether polyphenols exert compartment-specific effects and to define the underlying mechanism of action.

Although higher polyphenol intake was associated with fewer head abnormalities and more tail abnormalities, the clinical significance of these changes remains uncertain. The observed normal form% values were within ranges typically reported in healthy men, and motility was unaffected. Given the limited predictive value of isolated morphology measures for fertility outcomes, future prospective studies with reproductive endpoints are needed to clarify the clinical impact of these findings.

Taken together, these findings highlight the complexity of establishing a clear, causal relationship between dietary polyphenol intake and male fertility. Factors such as individual variability in absorption and metabolism, differences in gut microbiota, environmental exposures, and measurement inconsistencies across studies likely contribute to the observed heterogeneity.

This study presents some limitations. Given the typically poor bioaccessibility and variable bioavailability of dietary polyphenols in their native form, influenced by structural characteristics, individual microbiota composition, and metabolic conversion into numerous phenolic metabolites with diverse effects. Since we did not evaluate blood levels, dietary intake assessments may not accurately reflect true physiological exposure or biological activity. Thus, the associations observed in our study should be interpreted with caution. A polyphenol-specific, validated food frequency questionnaire and biomarker-based approaches are needed in future studies. Additionally, the relatively small sample size and cross-sectional design limit the generalizability and causal interpretation of the results.

## 5. Conclusions

In conclusion, our findings highlight a complex relationship between total dietary polyphenol intake, flavonoids and lignans, and sperm morphology, particularly with reduced sperm head abnormalities, but increased abnormalities in the sperm tail. Despite these associations, no significant relationship with overall sperm motility or vitality was observed, possibly due to compensatory mechanisms or the mild nature of the tail abnormalities that do not impair functional performance under standard assessments. This dual pattern emphasizes the complex role of polyphenols in male reproductive health and emphasizes the importance of considering both compound type and dosage, as well as employing sensitive functional evaluations. Although our results are not sufficient to support specific dietary recommendations, they suggest that polyphenols may have a role in maintaining certain aspects of semen quality. Future longitudinal studies and clinical trials incorporating fertilization outcomes are essential to clarify the mechanisms by which polyphenol-rich diets and specific polyphenol classes can influence sperm quality and reproductive potential, ultimately guiding nutritional strategies to support them.

## Figures and Tables

**Table 1:** Characteristics of the included participants.

Variables	Participants (n = 106)
General characteristics	
Age (years)	24.7 (4.7)
Weight (kg)	75.0 (10.7)
BMI (kg/m^2^)	23.8 (3.1)
Waist circumference (cm)	81.4 (8.1)
Systolic blood pressure (mmHg)	127.7 (11.6)
Diastolic blood pressure (mmHg)	72.4 (8.1)
Dietary polyphenol intake	
Total polyphenols intake (mg/day)	404.7 [296.0–529.2]
Flavonoids (mg/day)	244.6 [149.2–356.0]
Phenolic Acids (mg/day)	93.0 [69.1–140.4]
Stilbenes (mg/day)	0.02 [0.0–0.2]
Lignans (mg/day)	0.3 [0.2–0.6]
Other Polyphenols (mg/day)	47.0 [25.0–75.5]
Biochemical variables	
Fasting plasma glucose (mg/dL)	86 [82.3–92.8]
Total cholesterol (mg/dL)	168 [150.0–187.8]
HDL-c (mg/dL)	56 [50.0–64.8]
LDL-c (mg/dL)	95 [77.0–106.0]
VLDL-c (mg/dL)	13 [11.0–18.0]
Triglycerides (mg/dL)	66 [55.3–89.5]
Fasting plasma insulin (mcUI/mL)	5.5 [2.9–7.7]
C-reactive protein (mg/dL)	0.2 [0.2–0.2]
Sperm parameters	
pH	8 [8.0–8.5]
Volume (mL)	3 [2.0–4.5]
Total sperm (×10^6^)	72.1 [27.2–122.5]
Sperm concentration (×10^6^/mL)	24.1 [10.9–42.0]
Vitality (%)	79.3 [72.70–84.7]
Total motility (%)	66.5 [48.2–74.8]
Progressive motility (%)	47.3 [29.2–57.5]
Non-progressive motility (%)	11.8 [8.2–15.5]
Immotility (%)	33.6 [25.2–50.9]
Normal form (%)	6.4 [5.2–7.9]
Abnormality in the sperm head (%)	53.8 [42.3–66.3]
Abnormality in the mid-piece (%)	11.4 [8.1–15.0]
Abnormality in the principal piece (%)	12.7 [5.0–29.1]
Combined abnormality (%)	8.2 [6.5–13.39]
Nutrients intake	
Total energy intake (kcal/d)	2495.1 (613.6)
Carbohydrate intake (% E)	43.6 (6.5)
Protein intake (% E)	17.2 (3.4)
Fat intake (% E)	36.9 (6.2)
Monounsaturated fatty acids (% FE)	39.6 (6.8)
Polyunsaturated fatty acids (% FE)	12.2 (4.0)
Saturated fatty acids (% FE)	31.5 (6.2)
Dietary fiber intake (g/d)	21.5 (9.4)
Alcohol intake (g/d)	9.5 (15.1)

Data are presented as the mean (SD) and median [P25–P75]. BMI: body mass index, E: energy, FE: fat energy, HDL-c: high-density lipoprotein cholesterol, LDL-c: low-density lipoprotein cholesterol, and VLDL-c: very low-density lipoprotein cholesterol.

**Table 2:** Associations between the total and subclass-specific polyphenol intake and semen quality parameters.

Sperm Parameters	Total Polyphenols Intake					
	Tertile 1 (n = 35)	Tertile 2 (n = 35)	Tertile 3 (n = 36)	*P*-trend	β coefficient (95% CI)	*p*-value
Total polyphenol intake (mg/day)	260.82 [186.61–292.48]	404.75 [356.19–449.33]	671.75 [535.86–783.21]			
pH	Ref.	−0.001 (−0.009, 0.006)	0.003 (−0.005, 0.012)	0.37	0.005 (−0.010, 0.022)	0.50
Volume (mL)	Ref.	−0.024 (−0.170, 0.122)	−0.065 (−0.224, 0.094)	0.41	−0.075 (−0.377, 0.226)	0.62
Total sperm (×10^6^)	Ref.	0.077 (−0.199, 0.354)	0.049 (−0.252, 0.351)	0.41	0.310 (−0.257, 0.877)	0.28
Sperm concentration (×10^6^/mL)	Ref.	0.074 (−0.187, 0.336)	0.060 (−0.224, 0.345)	0.72	0.326 (−0.209, 0.863)	0.23
Vitality (%)	Ref.	0.035 (−0.013, 0.084)	0.025 (−0.027, 0.079)	0.42	0.043 (−0.057, 0.145)	0.40
Total motility (%)	Ref.	0.114 (−0.030, 0.258)	−0.040 (−0.197, 0.117)	0.45	0.002 (−0.302, 0.308)	0.99
Progressive motility (%)	Ref.	0.791 (−0.065, 1.647)	0.224 (−0.707, 1.156)	0.82	0.987 (−0.797, 2.772)	0.28
Non-progressive motility (%)	Ref.	−0.154 (−0.718, 0.408)	0.007 (−0.606, 0.620)	0.92	0.059 (−1.102, 1.221)	0.92
Immotility (%)	Ref.	−0.007 (−0.121 0.106)	0.075 (−0.047, 0.199)	0.19	0.069 (−0.166, 0.305)	0.56
Normal form (%)	Ref.	0.001 (−0.086, 0.088)	0.075 (−0.019, 0.171)	0.09	0.125 (−0.056, 0.306)	0.18
Abnormality in the head (%)	Ref.	−0.047 (−0.111, 0.016)	−0.084 (−0.153, −0.014)	0.02	−0.159 (−0.290, −0.027)	0.02
Abnormality in the mid-piece (%)	Ref.	0.024 (−0.090, 0.140)	0.054 (−0.070, 0.179)	0.39	0.032 (−0.205, 0.270)	0.788
Abnormality in the principal piece (%)	Ref.	0.147 (−0.084, 0.379)	0.375 (0.123, 0.627)	0.004	0.651 (0.170, 1.132)	0.01
Combined abnormality (%)	Ref.	−0.190 (−0.334, −0.047)	−0.088 (−0.244, 0.067)	0.44	−0.136 (−0.441, 0.168)	0.38
Sperm parameters	Lignans					
Tertiles	Tertile 1 (n = 37)	Tertile 2 (n = 34)	Tertile 3 (n = 35)	*P*-trend	β coefficient (95% CI)	*p*-value
Lignan intake (mg/day)	0.11 [0.09–0.16]	0.31 [0.23–0.40]	0.84 [0.61–1.12]			
pH	Ref.	−0.001 (−0.008, 0.007)	0.003 (−0.005, 0.012)	0.36	0.001 (−0.005, 0.006)	0.80
Volume (mL)	Ref.	−0.100 (−0.247, 0.046)	−0.158 (−0.319, 0.003)	0.08	−0.056 (−0.161, 0.048)	0.29
Total sperm (×10^6^)	Ref.	−0.123 (−0.406, 0.159)	−0.082 (−0.392, 0.227)	0.77	−0.073 (−0.272, 0.125)	0.47
Sperm concentration (×10^6^/mL)	Ref.	0.038 (−0.228, 0.306)	0.111 (−0.182, 0.405)	0.44	0.004 (−0.183, 0.193)	0.96
Vitality (%)	Ref.	0.017 (−0.033, 0.067)	0.010 (−0.044, 0.065)	0.44	−0.005 (−0.040, 0.030)	0.77
Total motility (%)	Ref.	0.085 (−0.064, 0.236)	0.060 (−0.105, 0.225)	0.68	0.025 (−0.081, 0.132)	0.63
Progressive motility (%)	Ref.	0.443 (−0.444, 1.330)	0.298 (−0.676, 1.272)	0.74	0.269 (−0.355, 0.894)	0.40
Non-progressive motility (%)	Ref.	0.393 (−0.176, 0.963)	0.413 (−0.211, 1.039)	0.31	0.129 (−0.275, 0.534)	0.53
Immotility (%)	Ref.	0.010 (−0.106, 0.128)	−0.019 (−0.148, 0.109)	0.68	0.003 (−0.078, 0.086)	0.93
Normal form (%)	Ref.	0.058 (−0.031, 0.149)	0.025 (−0.073, 0.124)	0.91	−0.008 (−0.072, 0.056)	0.80
Abnormality in the head (%)	Ref.	−0.030 (−0.096, 0.035)	−0.075 (−0.147, −0.003)	0.04	−0.042 (−0.088, 0.004)	0.08
Abnormality in the mid-piece (%)	Ref.	−0.054 (−0.172, 0.062)	−0.019 (−0.148, 0.109)	0.10	−0.063 (−0.145, 0.018)	0.13
Abnormality in the principal piece (%)	Ref.	0.116 (−0.125, 0.359)	0.277 (0.011, 0.543)	0.04	0.162 (−0.008, 0.333)	0.06
Combined abnormality (%)	Ref.	0.008 (−0.141, 0.159)	0.107 (−0.057, 0.272)	0.15	0.056 (−0.050, 0.162)	0.30
Sperm parameters	Stilbenes					
	Tertile 1 (n = 36)	Tertile 2 (n = 36)	Tertile 3 (n = 34)	*P*-trend	β coefficient (95% CI)	*p*-value
Stilbene intake (mg/day)	0 [0–0.00001]	0.02 [0–0.04]	0.95 [0.22–2.56]			
pH	Ref.	−0.005 (−0.013, 0.001)	0 (−0.009, 0.008)	0.43	−0.001 (−0.001, 0)	0.18
Volume (ml)	Ref.	0.189 (0.058, 0.321)	−0.004 (−0.156, 0.147)	0.12	0.007 (−0.007, 0.022)	0.30
Total sperm (×10^6^)	Ref.	0.184 (−0.071, 0.440)	−0.100 (−0.397, 0.195)	0.12	0.003 (−0.024, 0.032)	0.79
Sperm concentration (×10^6^/mL)	Ref.	−0.022 (−0.270, 0.225)	−0.092 (−0.378, 0.194)	0.52	−0.004 (−0.031, 0.022)	0.74
Vitality (%)	Ref.	−0.022 (−0.270, 0.225)	−0.092 (−0.378, 0.194)	0.13	−0.002 (−0.007, 0.002)	0.36
Total motility (%)	Ref.	−0.036 (−0.175, 0.102)	−0.108 (−0.269, 0.052)	0.21	−0.007 (−0.022, 0.007)	0.31
Progressive motility (%)	Ref.	−0.537 (−1.351, 0.276)	−0.717 (−1.659, 0.223)	0.32	−0.072 (−0.159, 0.014)	0.10
Non-progressive motility (%)	Ref.	−0.311 (−0.833, 0.209)	0.283 (−0.319, 0.886)	0.08	−0.003 (−0.060, 0.054)	0.91
Immotility (%)	Ref.	−0.015 (−0.123, 0.093)	0.045 (−0.079, 0.170)	0.32	0.002 (−0.009, 0.013)	0.72
Normal form (%)	Ref.	−0.044 (−0.128, 0.039)	−0.053 (−0.150, 0.042)	0.50	−0.006 (−0.015, 0.002)	0.17
Abnormality in the head (%)	Ref.	−0.008 (−0.070, 0.053)	−0.038 (−0.109, 0.033)	0.28	−0.003 (−0.010, 0.003)	0.31
Abnormality in the mid-piece (%)	Ref.	0.004 (−0.103, 0.112)	0.086 (−0.037, 0.211)	0.12	0.004 (−0.010, 0.003)	0.50
Abnormality in the principal piece (%)	Ref.	−0.019 (−0.248, 0.208)	0.102 (−0.161, 0.366)	0.32	0.008 (−0.010, 0.003)	0.50
Combined abnormality (%)	Ref.	−0.010 (−0.146, 0.124)	0.175 (0.018, 0.332)	0.01	0.011 (−0.003, 0.026)	0.14
Sperm parameters	Flavonoids					
	Tertile 1 (n = 35)	Tertile 2 (n = 35)	Tertile 3 (n = 36)	*P*-trend	β coefficient (95% CI)	*p*-value
Flavonoid intake (mg/day)	124.56 [86.94–147.83]	244.10 [213.67–283.93]	412.56 [355.98–570.56]			
pH	Ref.	−0.001 (−0.008, 0.007)	0.003 (−0.005, 0.011)	0.37	0.002 (−0.008, 0.013)	0.69
Volume (ml)	Ref.	0.018 (−0.122, 0.158)	−0.003 (−0.145, 0.150)	0.10	−0.079 (−0.277, 0.117)	0.42
Total sperm (×10^6^)	Ref.	0.201 (−0.059, 0.463)	0.160 (−0.115, 0.436)	0.32	0.189 (−0.182, 0.561)	0.32
Sperm concentration (×10^6^)	Ref.	0.087 (−0.161, 0.337)	0.084 (−0.179, 0.347)	0.56	0.211 (−0.140, 0.562)	0.24
Vitality (%)	Ref.	0.023 (−0.023, 0.070)	0.008 (−0.040, 0.058)	0.82	0.003 (−0.063, 0.070)	0.92
Total motility (%)	Ref.	0.040 (−0.099, 0.181)	−0.044 (−0.192, 0.103)	0.47	−0.062 (−0.261, 0.137)	0.54
Progressive motility (%)	Ref.	0.619 (−0.204, 1.442)	0.365 (−0.502, 1.233)	0.50	0.516 (−0.655, 1.688)	0.38
Non-progressive motility (%)	Ref.	−0.175 (−0.711, 0.361)	0.058 (−0.506, 0.624)	0.75	0 (−0.760, 0.761)	0.10
Immotility (%)	Ref.	−0.001 (−0.109, 0.106)	0.084 (−0.029, 0.198)	0.11	0.082 (−0.071, 0.236)	0.29
Normal form (%)	Ref.	0.043 (−0.039, 0.126)	0.088 (0.0, 0.175)	0.049	0.091 (−0.027, 0.210)	0.13
Abnormality in the head (%)	Ref.	−0.025 (−0.086, 0.034)	−0.078 (−0.142, −0.014)	0.01	−0.086 (−0.173, 0.001)	0.05
Abnormality in the mid-piece (%)	Ref.	−0.031 (−0.139, 0.077)	0.053 (−0.061, 0.168)	0.28	0.007 (−0.147, 0.163)	0.92
Abnormality in the principal piece (%)	Ref.	0.098 (−0.124, 0.321)	0.305 (0.069, 0.540)	0.01	0.371 (0.053, 0.689)	0.02
Combined abnormality (%)	Ref.	−0.124 (−0.264, 0.014)	−0.085 (−0.232, 0.062)	0.34	−0.129 (−0.328, 0.069)	0.20

The total and subclass-specific polyphenol intake are shown as medians [P25–P75]. The results are shown as β coefficients and their 95% confidence interval (CI). Both the exposure and outcome were log-transformed. Coefficients represent the percent change in the outcome associated with 1% change in the exposure. Linear regression models were adjusted for age, alcohol intake, body mass index, total energy intake, protein intake, fiber intake, carbohydrate intake, cholesterol intake, and fatty acids intake (monounsaturated, polyunsaturated, and saturated).

## Data Availability

The data supporting the findings are available from the corresponding author upon reasonable request.

## Supplementary Materials

The following supporting information can be downloaded at https://www.mdpi.com/article/10.3390/nu17172785/s1, Table S1: Association between the intake of other polyphenols and phenolic acids with semen quality parameters.

## Abbreviations

The following abbreviations are used in this manuscript:

WHO	World Health Organization
BMI	Body Mass Index
IQR	Interquartile Range
LDL	Low-Density Lipoprotein
VLDL	Very Low-Density Lipoprotein
HDL	High-Density Lipoprotein
E	Energy
FE	Fat Energy
TPI	Total Polyphenols Intake
PA	Phenolic Acids
OPP	Other Polyphenols
DASH	Dietary Approaches to Stop Hypertension
EGFR	Epidermal Growth Factor Receptor
