# Supplementary material for: Association Between Dietary Polyphenol Intake and Semen Quality: Insights from the FERTINUTS Study
Source: Nutrients. 2025 Aug 27;17(17):2785. doi: 10.3390/nu17172785 (PMC12430331; doi:10.3390/nu17172785)
Supplement: Supplementary file 1 [file nutrients-17-02785-s001.zip › nutrients-3810573-supplementary.pdf]

Supplementary file

Supplementary Table S1: Association between the intake of other polyphenols and phenolic acids with semen quality parameters.

Sperm parameters	Other polyphenols					
Other polyphenols intake (mg/day)	Tertile 1 (n=35) 124.56 [86.94 - 147.83]	Tertile 2 (n=35) 244.10 [213.67 - 283.93]	Tertile 3 (n=36) 412.56 [355.98 - 570.56]	**P-trend**	**β coefficient (95% CI)**	**P-value**
pH	Ref	0.002 (-0.005, 0.010)	0.004 (-0.003, 0.013)	0.26	0.012 (0.001, 0.022)	**0.02 ***
Volume (mL)	Ref	0.056 (-0.090, 0.204)	0.042 (-0.109, 0.195)	0.64	-0.038 (-0.228, 0.151)	0.69
Total sperm ($x10^6$)	Ref	-0.096 (-0.374, 0.181)	0.019 (-0.268, 0.307)	0.80	-0.015 (-0.374, 0.343)	0.93
Concentration ($x10^6$/mL)	Ref	-0.122 (-0.374, 0.181)	0.029 (-0.242, 0.300)	0.71	0.048 (-0.291, 0.387)	0.79
Vitality (%)	Ref	-0.007 (-0.057, 0.042)	0.003 (-0.047, 0.055)	0.85	0.009 (-0.054, 0.073)	0.78
Total Motility (%)	Ref	-0.057 (-0.206, 0.091)	-0.004 (-0.158, 0.149)	0.95	-0.030 (-0.222, 0.161)	0.75
Progressive motility (%)	Ref	-0.656 (-1.523, 0.210)	-0.435 (-1.334, 0.462)	0.95	-0.548 (-1.672, 0.575)	0.34
Non-progressive motility (%)	Ref	-0.395 (-0.956, 0.166)	-0.253 (-0.835, 0.328)	0.49	-0.326 (-1.054, 0.400)	0.37
Immotility (%)	Ref	-0.007 (-0.122, 0.108)	0.006 (-0.113, 0.126)	0.89	0.006 (-0.142, 0.155)	0.93
Normal form (%)	Ref	-0.007 (-0.095, 0.079)	0.077 (-0.013, 0.168)	0.07	0.089 (-0.024, 0.203)	0.12
Abnormality in the head (%)	Ref	-0.027 (-0.092, 0.037)	-0.068 (-0.135 -0.001)	0.07	0.089 (-0.024, 0.203)	0.06
Abnormality in the mid-piece (%)	Ref	0.056 (-0.058, 0.172)	0.030 (-0.089, 0.150)	0.70	0.096 (-0.051, 0.244)	0.12
Abnormality in the principal piece (%)	Ref	0.122 (-0.118, 0.364)	0.154 (-0.096, 0.405)	0.25	0.243 (-0.066, 0.553)	0.12
Combined abnormality (%)	Ref	-0.019 (-0.168, 0.130)	-0.027 (-0.182, 0.127)	0.25	0.243 (-0.066, 0.553)	0.53
**Sperm parameters**	**Phenolic acids**					
**Phenolic acids intake (mg/day)**	**Tertile 1** (n=35) 63.60 [51.16 - 68.78]	**Tertile 2** (n=35) 93.03 [86.36 - 99.20]	**Tertile 3** (n=36) 168.71 [142.67 - 193.22]	**P-trend**	**β coefficient (95% CI)**	**P-value**
pH	Ref	-0.003 (-0.011, 0.004)	-0.004 (-0.012, 0.004)	0.47	-0.002 (-0.0181, 0.014)	0.78
Volume (mL)	Ref	0.155 (0.011, 0.298)	0.103 (-0.046, 0.253)	0.49	0.052 (-0.237, 0.342)	0.72
Total sperm ($x10^6$)	Ref	0.131 (-0.144 0.407)	0.066 (-0.222, 0.355)	0.89	0.150 (-0.396, 0.697)	0.59
Concentration ($x10^6$/mL)	Ref	-0.011 (-0.273, 0.250)	-0.044 (-0.319, 0.229)	0.73	0.105 (-0.412, 0.623)	0.69

Vitality (%)	Ref	0.034 (-0.014, 0.083)	0.037 (-0.013, 0.088)	0.26	0.084 (-0.011, 0.180)	0.08
Total Motility (%)	Ref	0.130 (-0.015, 0.276)	0.093 (-0.059, 0.245)	0.51	0.225 (-0.064, 0.514)	0.13
Progressive motility (%)	Ref	0.560 (-0.304, 1.424)	0.367 (-0.538, 1.272)	0.69	1.228 (-0.475, 2.932)	0.16
Non-progressive motility (%)	Ref	0.228 (-0.332, 0.789)	0.292 (-0.295, 0.880)	0.41	0.043 (-1.070, 1.158)	0.94
Immotility (%)	Ref	-0.110 (-0.223, 0.001)	-0.059 (-0.177, 0.058)	0.73	-0.090 (-0.316, 0.135)	0.43
Normal form (%)	Ref	-0.070 (-0.158, 0.017)	-0.057 (-0.149, 0.034)	0.44	0.015 (-0.160, 0.191)	0.86
Abnormality in the head (%)	Ref	0.074 (0.011, 0.136)	-0.005 (-0.071, 0.060)	0.24	-0.050 (-0.180, 0.078)	0.44
Abnormality in the mid-piece (%)	Ref	0.003 (-0.111, 0.118)	0.043 (-0.076, 0.164)	0.41	0.082 (-0.145, 0.309)	0.47
Abnormality in the principal piece (%)	Ref	-0.286 (-0.519, -0.054)	-0.027 (-0.270, 0.215)	0.44	0.147 (-0.330, 0.625)	0.54
Combined abnormality (%)	Ref	0.056 (-0.091, 0.203)	0.103 (-0.050, 0.257)	0.21	0.193 (-0.098, 0.484)	0.19

Other polyphenol and phenolic acids intake are shown as medians [P25 - P75]. The results are shown as β coefficients and their 95% confidence intervals (CIs). Both the exposure and outcome were log-transformed. Coefficients represent the percent change in the outcome associated with a 1% change in the exposure. Linear regression models were adjusted for age, alcohol intake, body mass index, total energy intake, protein intake, fibre intake, carbohydrate intake, cholesterol intake, and fatty acids intake (monounsaturated, polyunsaturated, and saturated).
